# ReUse: REgressive Unet for Carbon Storage and Above-Ground Biomass Estimation

**DOI:** 10.3390/jimaging9030061

**Published:** 2023-03-07

**Authors:** Antonio Elia Pascarella, Giovanni Giacco, Mattia Rigiroli, Stefano Marrone, Carlo Sansone

**Affiliations:** 1 Department of Electrical Engineering and Information Technology (DIETI), University of Naples Federico II, Via Claudio 21, 80125 Naples, Italy; 2 Latitudo 40, Via Emanuele Gianturco 31/c, 80146 Naples, Italy

**Keywords:** U-Net, carbon storage, above-ground biomass, remote sensing, deep learning, CNN, Sentinel-2, ESA CCI Biomass project

## Abstract

The United Nations Framework Convention on Climate Change (UNFCCC) has recently established the Reducing Emissions from Deforestation and forest Degradation (REDD+) program, which requires countries to report their carbon emissions and sink estimates through national greenhouse gas inventories (NGHGI). Thus, developing automatic systems capable of estimating the carbon absorbed by forests without in situ observation becomes essential. To support this critical need, in this work, we introduce ReUse, a simple but effective deep learning approach to estimate the carbon absorbed by forest areas based on remote sensing. The proposed method’s novelty is in using the public above-ground biomass (AGB) data from the European Space Agency’s Climate Change Initiative Biomass project as ground truth to estimate the carbon sequestration capacity of any portion of land on Earth using Sentinel-2 images and a pixel-wise regressive UNet. The approach has been compared with two literature proposals using a private dataset and human-engineered features. The results show a more remarkable generalization ability of the proposed approach, with a decrease in Mean Absolute Error and Root Mean Square Error over the runner-up of 16.9 and 14.3 in the area of Vietnam, 4.7 and 5.1 in the area of Myanmar, 8.0 and 1.4 in the area of Central Europe, respectively. As a case study, we also report an analysis made for the Astroni area, a World Wildlife Fund (WWF) natural reserve struck by a large fire, producing predictions consistent with values found by experts in the field after in situ investigations. These results further support the use of such an approach for the early detection of AGB variations in urban and rural areas.

## 1. Introduction

An accurate assessment of forest above-ground biomass (AGB), which in this work is defined as the mass expressed as the oven-dry weight of the woody parts (stem, bark, branches, and twigs) of all living trees (excluding stump and roots) and their related carbon stock, is essential for the sustainable management of forests. Recently, the United Nations Framework Convention for Climate Change (UNFCCC) established the Reducing Emissions from Deforestation and forest Degradation (REDD+) program, which requires countries to report their carbon emissions and sink estimates through national greenhouse gas inventories (NGHGI) [1,2]. Furthermore, Sustainable Development Goal 15 aims to “protect, restore and promote the sustainable use of terrestrial ecosystems, sustainably manage forests, combat desertification, halt and reverse land degradation and halt biodiversity loss” [3]. Subsequently, it is paramount to conduct an explicit investigation into the methods and procedures for quantifying carbon sinks. Generally, the above-ground dry biomass holds about 50% of carbon; as such, a friction factor of 0.5 is commonly used for converting AGB into carbon concentration [4,5].

There are two basic approaches to obtaining biomass estimation: traditional field-based and remote sensing (RS) methods. There is no doubt that traditional methods are more accurate [6]; still, they are also much more time consuming, laborious, and challenging to implement in inaccessible areas, as well as being destructive in nature [7]. An increasingly investigated solution is to rely on images acquired by satellites and/or drones. Among all of them, the Sentinel-2 satellite system, launched on 23 June 2015 by the European Space Agency (ESA) and equipped with a multi-spectral instrument (MSI), presents a high potential for applications in land management, the agricultural industry (food security), forestry (AGB) disaster control, and humanitarian relief operations [8]. Sentinel-2 is a polar-orbiting satellite system comprising two satellites, each carrying an MSI characterized by a 290 km swath width, offering a multi-purpose design of 13 spectral bands traversing from visible and near-infrared (NIR) wavelengths to short-wave infrared wavelengths at refined (10, 20 m) and coarse (60 m) spatial resolutions. Furthermore, the presence of four bands within the red-edge region, centred at 705 (band 5), 740 (band 6), 783 (band 7), and 865 nm (band 8a), gives the satellite system the potential for mapping various vegetation characteristics [9].

Most of the approaches so far developed make use of classical machine learning models (such as SVM, random forest, etc.) to estimate above-ground biomass and the related carbon stock, leveraging expert-made features to be extracted from Sentinel-2 images [10,11,12,13,14]. More recently, some works [15,16] are investigating the use of convolutional neural networks (CNN) to estimate AGB using the commercial satellite Worldview-2 and visible spectrum images captured by an unmanned aerial vehicle. In all the reported examples, the AGB target variable is always collected by field measurements (i.e., relying on data collected by experts physically located on the target terrain). This characteristic strongly limits the usability of AI-based AGB automatic estimators in a real context, such as the continual analysis of the impact of natural disasters, as well as monitoring the effectiveness of environmental policies, which requires the reliable acquisition of the ground truth on wide and heterogeneous landscapes.

To cope with this need, in this work, we propose to use the open data released by the ESA Climate Change Initiative (CCI) BIOMASS project as the ground truth for AGB. This, together with the images acquired by the Sentinel-2 system, allows, unlike field measurements, the acquisition of AGB on a global scale. To the best of our knowledge, this is the first time that this data combination has been used for AGB estimation. The rest of the paper is as follows: Section 2 reports a detailed review of the approaches so far proposed for AGB estimation from satellite images; Section 3 details the proposed approach, as well as the considered dataset, competition, and experimental setup; Section 4 reports the obtained results and a case study analysis performed on a WWF natural reserve struck by a severe wildfire; finally, Section 5 draws some conclusions.

## 2. Related Works

The literature on remote sensing for vegetation detection is wide. However, above-ground biomass detection is a different and more crucial task, especially when needed for the detailed estimation of other indicators, such as the carbon sequestration ability of a portion of land. Thus, in this section, we focus only on works aimed at estimating above-ground biomass and carbon storage from Sentinel-2 images.

In [13], the authors attempted to examine the prospects of Sentinel-2 spectral data for quantifying the carbon stock in a reforested urban landscape, using a random forest and, as predictors, 10 Sentinel bands plus 15 spectral indices that summarize the spectral content without taking into account in any way the spatial correlations typical of an image. Similarly, in [10], the authors applied Sentinel-2 satellite images combined with field-measured biomass using random forest (RF) to estimate above-ground biomass in Yok Don National Park, Vietnam. A total of 132 spectral and texture variables were extracted from the Sentinel-2 images; the gray-level co-occurrence matrix (GLCM) method [17] was used to compute the texture variables.

In [12], Sentinel-2 performance was evaluated for a buffer-zone community forest in Parsa National Park, Nepal, using field-based AGB as a dependent variable, as well as spectral band values and spectral-derived vegetation indices as independent variables in the random forest algorithm; in this study, no features were extracted from the spatial dimensions, but indicators were only extracted from the spectral dimension of the input tensor. Spectral bands, vegetation indices (VIs), and texture variables derived from processed Sentinel-2 data and topographic parameters have been used in [14] to create statistical links with field-based AGB by implementing random forest and stochastic gradient boosting (SGB) algorithms. The gray-level co-occurrence matrix method [17] and wavelet decomposition were applied using the first principal component of the Sentinel-2 multispectral tensor.

In [18], to estimate the AGB from remotely sensed data and parametric and non-parametric methods, multiple regression (MR), k-nearest neighbour (kNN), random forest, and the multi-layer perceptron, which performed best among the various methods, were applied to a single Sentinel-2 image using spectral bands and derived indices. Similarly, in [19], the authors explored the capability of the spectral and texture features of the Sentinel-2 multispectral instrument (MSI) for modeling grassland AGB using the random forest (RF) and extreme gradient boosting (XGBoost) algorithms in Shengjin Lake wetland (a Ramsar site), showing that the RF and XGBoost models had a robust and efficient performance and that the introduction of eight gray-level co-occurrence matrix (GLCM) textures moderately improved the accuracy of modeling AGB. The texture is also the core of [20], in which texture metrics were derived based on different working window sizes (3 × 3, 5 × 5, 7 × 7, and 9 × 9), and the results were compared with those obtained using raw traditional bands (Band 2, 3, 4, 8, 11 and 12), raw traditional and red-edge bands (Band 5, 6, 7 and 8A), and red-edge bands only: the use of texture with a 7 × 7 window size and vegetation indices (VIs) yielded higher biomass estimates.

In [21], the authors proposed an innovative and dynamic architecture based on the generative neural network that extracts target-oriented generative features for forest prediction AGB using satellite data. The architecture exploits its generative capacity to produce variables in a latent space to predict AGB, exploiting only the spectral dimension and not the spatial correlations of the images. In the same year, in [22], the authors presented an automated machine learning (AutoML) framework for modeling, evaluating, and stacking multiple base models for AGB prediction. This work incorporates a hyperparameter optimization procedure for automatically extracting targeted features from multi-temporal Sentinel-2 data, minimizing human bias. Furthermore, in this context, the automatic feature extraction took into account only the spectral dimension. Finally, a recent work [23] mainly discusses three non-parametric models: the artificial neural network (ANN), random forests, and, in particular, the quantile regression neural network (QRNN), using spectral index and texture features as variables.

These works use machine learning techniques combined with intensive feature extraction, with some focusing only on the spectral dimension and others involving both the spatial and spectral dimensions. In addition to the used features, some works [21,22] also exploited generative networks and AutoMl pipelines to minimize the human bias in the feature extraction phase. Moreover, as for other image-processing-related domains, researchers [15,16] are also working on the use of convolutional neural networks (CNNs), which are designed to produce numerical values for AGB prediction (one for each input image), using input from commercial satellites, such as Worldview-2, or visible spectrum images captured by an unmanned aerial vehicles instead of Sentinel-2 open data.

As far as we know, no deep learning approach relying on fully convolutional architecture (such as UNet) trained on Sentinel-2 multi-spectral images has been proposed so far to predict AGB and carbon storage. In this work, we cope with this lack with the aim of introducing a new approach that embeds feature extraction within the network to produce, not a simple numerical value for the whole input image (as in the case of CNNs), but a mask of numerical values associating the AGB value to each pixel in the input image. This combines very fine-grained estimation and wide computational advantages, together with a greater generalization ability, especially in the case of learning over large and variegate geographic areas. Moreover, all the reported works, including recent ones [24] designed to provide an in-depth analysis of forests and individual trees’ carbon storage estimation, use AGB field measurements as the target variable and/or require input data for which field surveys are necessary. Instead, in our work, we rely on the ESA public data for AGB. The aim is to show that the combined use of a regressive UNet network with public data (Sentinel-2 and ESA AGB) can help monitor carbon content in forest areas at a global scale in a close to real-time manner (i.e., as soon as a new image is available from the Sentinel-2 system).

## 3. Materials and Methods

In this paper, we introduce a regressive UNet trained on the public above-ground biomass data from the European Space Agency’s Climate Change Initiative Biomass project as ground truth to estimate the carbon sequestration capacity of any portion of land on Earth using Sentinel-2 images, comparing its performance against two literature proposals [10,14] on their respective study areas. Section 3.1 introduces the proposed approach, describing ideas and motivations. Section 3.2 details the experimental setup, as well as the considered competitors. Finally, Section 3.3 describes the considered dataset, focusing on data acquisition and pre-processing.

### 3.1. Regressive UNet

In the following study, we introduce ReUse, a UNet network trained to carry out a pixel-wise regression task to map Sentinel-2 images into AGB rasters. The UNet was developed by [25] for biomedical semantic segmentation. In the original proposal, the architecture contains two paths. The first is a contraction path (also known as the encoder) designed to capture the context in the image. Several structures are possible; however, they usually all involve sequences of convolutional and max pooling layers. The second path is a symmetric expanding path (also known as the decoder) designed to produce a pixel-wise prediction using transposed convolutions. These two paths are connected by some skipping connections, designed to improve the localization ability of the network by combining the high-resolution features from the contracting path with the corresponding one in the expanding path. A final convolution layer can then be used to learn and assemble a more precise output based on this information.

**Figure 1:**
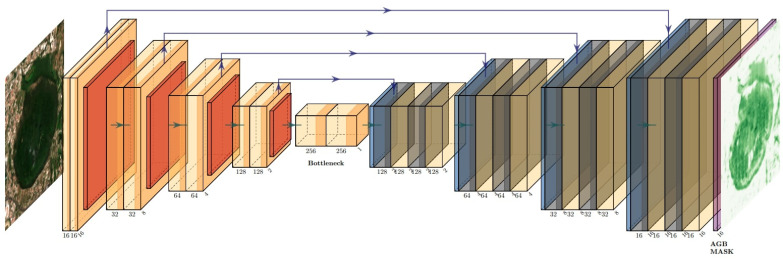
ReUse architecture for pixel-wise regression. The input is the Sentinel-2 image with dimensions (patch size, patch size, number of channels); the output is the AGB image with dimensions (patch size, patch size, 1).

The main difference we introduce to the original UNet architecture is that the network was trained not for performing semantic segmentation but to produce a pixel-wise regression map. This was obtained by omitting the softmax in the last layer, thus forcing the optimizer to minimize the loss function based on the actual values predicted by the network, directly comparing them with the AGB ground truth (Figure 1). To the best of our knowledge, this is the first time such an approach has been used to estimate AGB. The main advantage is that the proposed variant is able to extract both spatial and spectral features from the satellite multi-spectral images using an end-to-end paradigm. In particular, a patch-wise approach was used [26], dividing each Sentinel-2 input and AGB-raster into non-overlapping patches of 16 × 16 pixels.

### 3.2. Experimental Setup

One of the problems with the evaluation of remote sensing applications is the lack of a standard experimental protocol. Indeed, given the high spatial and temporal variability associated with images captured by satellite sensors, selecting different Earth zones and acquisition times can result in different levels of performance, making any comparison with other literature approaches less fair. To cope with this, we perform different evaluations on different Earth zones, comparing ReUse against two literature proposals [10,14] both leveraging a random forest [27] classifier trained on spatial and spectral features in an end-to-end paradigm. It is worth noting that in [14] the authors also use topographical parameters, such as altitude as a variable. However, as that data is not always available, in this work only Sentinel-2 data were used as input.

Comparisons between ReUse and the [10,14] approaches were made on study areas used by the latter, located in the Central Highlands of Vietnam and the Yinmar Forest (YM) located in the northern and central-eastern part of Myanmar, respectively. In [10], a total of 132 spectral and texture variables was extracted from the Sentinel-2 images; the gray-level co-occurrence matrix (GLCM) method [17] was used to compute mean, variance, homogeneity, contrast, dissimilarity, entropy, second moment, and correlation. In [14], principal component analysis (PCA) [28] was used to eliminate correlated information in satellite images and simultaneously reduce their dimensionality. The first principal component (PC1) was used for texture extraction. When extracting textural features, the gray-level co-occurrence matrix method [17] was used to compute mean, variance, homogeneity, contrast, dissimilarity, entropy, second moment, and correlation, and wavelet decomposition methods were also applied, considering their usefulness for the representation of relevant features [29]. The wavelet analysis produces four essential components: the approximation image, and horizontal detail, vertical detail, and diagonal detail images. The latter three are regarded as helpful textural measures. In particular, the Coiflect discrete wavelet function was chosen. Thus, based on the first principal component, a three-level decomposition strategy was implemented to generate nine detailed images as independent textural variables for AGB modeling. Finally, 2 types of textures derived from GLCM-based and wavelet analysis were included in the AGB modeling in combination with 11 spectral indices.

The proposed ReUse architecture has instead been trained using two different setups: one relying only on the raw Sentinel-2 bands, suitably normalized; the other leveraging a raw band together with textural and spectral indices, as in [10,14], respectively. Focusing on the latter setup, concerning the texture variables, the GLCM method was adopted to compute mean, variance, homogeneity, contrast, dissimilarity, entropy, second moment, and correlation, to which the nine detailed images obtained by applying the Coiflect wavelets have been added by adopting a three-level strategy. The texture variables were computed using the first principal component of the raw Sentinel bands. In this work, a 5×5 kernel was used to construct the GLCM-based features. Indeed, considering that training was conducted at a spatial resolution of 100 m (because this is the spatial resolution of ESA’s AGB data), such a kernel involves an area of 500 m by 500 m.

For all the considered approaches (including ours and competitors), the images have been rescaled to a spatial resolution of 100 m to match the ESA CCI Biomass Project AGB data. All the experiments have been run using an 8-fold cross-validation strategy, where each fold contains only patches associated with the same zone on Earth, while different folds refer to different portion of Earth (thus ensuring that there are no data leaks between different folds). For each iteration, 6 of the 8 folds were used for training, 1 for validation, and 1 for testing. The validation set was used to optimize the number of epochs for ReUse with an early stopping procedure [30] and the number of trees for the two competitors using two possible values: 250 or 500. In this study, concerning the early stopping procedure, training is stopped when the monitored validation loss has stopped improving after 35 epochs; the maximum number of epochs is set at 500. The optimizer used is Adam [31] with the default parameters. For the learning rate, following the approach introduced in [32], if no improvement in validation loss is seen for 25 epochs, the learning rate is reduced by a factor of 0.2. The Mean Absolute Error is used as a loss function for ReUse. The trees in the random forest are maximally grown, and the number of variables that each tree can choose at each split is equal to the square root of the number of features, as suggested by [33]. For reproducibility purposes, the source code is available at our GitHub repository (https://github.com/priamus-lab/ReUse, accessed on 1 March 2023).

### 3.3. Image Acquisition and Pre-Processing

Starting from the Global Dataset of above-ground biomass of the year 2018, version 3, of the ESA CCI BIOMASS Project, three study areas were downloaded and used separately to compare ReUse with the competitors [10,14]. The file “N20E100” contains the AGB of the study area of [10] in Vietnam, while the file “N30E90” contains the AGB of the study area of Yinmar (YM) Forest [14] in Myanmar. It is again emphasized that [10,14] use field AGB data, while in this work, public AGB data provided by ESA are used. The third study area cut from the “N60E00” file in Central Europe was used to compare the approaches in a western area and for the Astroni use case (Section 4.1). Table 1 shows the names of the downloaded AGB files in GeoTIFF format and the clipped areas of interest in well-known text (WKT), a text markup language for representing vector geometry objects. From the WKTs, it was possible to download the corresponding Sentinel-2 L2A satellite multispectral images acquired during available cloud-free days. The dates of the downloaded Sentinel-2 images of Vietnam, Myanmar, and Europe are 3rd April, 7th March, and 27th July 2018, respectively.

The Sentinel-2 satellite system acquires images with 13 spectral channels at variable spatial resolutions of 10, 20, and 60 m. This satellite system covers the red-edge region (i.e., b5, 6, 7, and 8A), strategically positioned in the electromagnetic spectrum with unique band settings critical for vegetation modeling [34]. For ReUse, bands 1, 9, and 10 were eliminated due their coarse spatial resolution, resulting in the use of only 10-band images. The values in the retrieved rasters are digital numbers (DN) that must be transformed into reflectance by dividing them by the quantification value. The quantification value in the Sentinel-2 product metadata is equal to 10,000 [35]. The infrastructure provided by the company Latitudo 40 was used to download and prepare the Sentinel-2 data described above. Concerning the AGB data, the dataset [36] comprises estimates of forest above-ground biomass for 2010 [37], 2017, and 2018. The estimates are derived from a combination of Earth observation data, depending on the year, from the Copernicus Sentinel-1 mission, Envisat’s ASAR instrument, and JAXA’s Advanced Land Observing Satellite (ALOS-1 and ALOS-2), along with additional information from Earth observation sources. The data have been produced as part of the European Space Agency’s Climate Change Initiative program by the Biomass CCI team. The mapping is at 100 m grid spacing with a target relative error of less than 20% where AGB exceeds 50 Mg/ha.

## 4. Results

As described in the previous section, the proposed ReUse approach (both versions) has been compared against two machine learning approaches [10,14] using random forest and leveraging both spatial and spectral dimensions during the feature-engineering phase. The study areas used are of Vietnam and Myanmar to compare ReUse with competitors in the areas in which these methodologies were initially designed. Moreover, we also consider an area in Central Europe to test the models on the territorial characteristics of a western country. Eight-fold cross-validation was used to estimate the error of the models. Table 2 presents the experiments for Vietnam, Myanmar, and Central Europe, highlighting that ReUse performs better than its competitors in terms of MAE, RMSE, and $R^{2}$.

**Figure 2:**
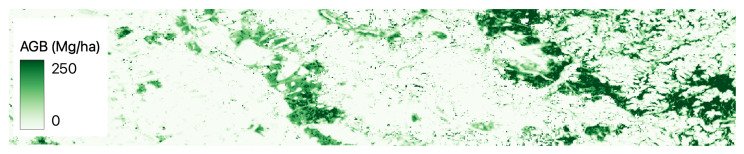
Base map of the test area in Central Europe.

**Figure 3:**
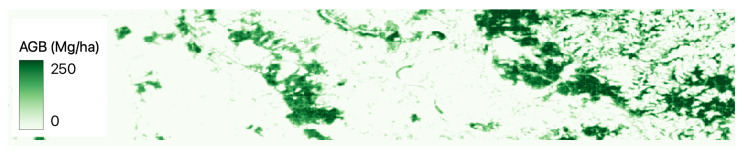
ReUse’s AGB predictions with raw bands in Central Europe.

**Figure 4:**
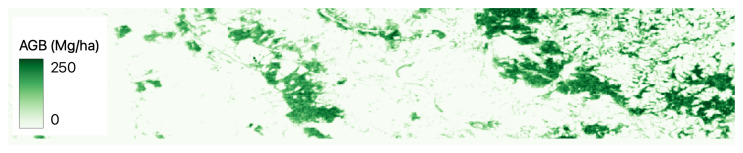
ReUse’s AGB predictions with raw bands and feature extraction in Central Europe.

**Figure 5:**
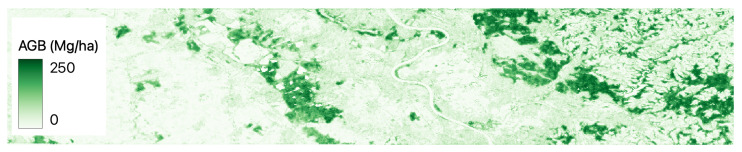
Predictions of AGB using the machine learning approach [10] in Central Europe.

**Figure 6:**
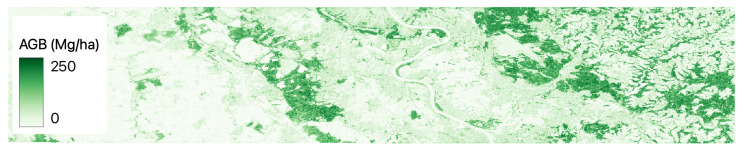
Predictions of AGB using the machine learning approach [14] in Central Europe.

Figure 2, Figure 3, Figure 4, Figure 5 and Figure 6 show a base map of the test area in Central Europe and the AGB predictions of the different approaches. It can be seen that the city areas where there is no greenery are appropriately set to 0, and the tree areas are all highlighted. In particular, our solutions (Figure 3 and Figure 4) show more marked differences between green and non-green areas than the classical machine learning approaches (Figure 5 and Figure 6). Furthermore, the experiments show that ReUse with feature extraction and 42 spectral indexes (as in [10]) in conjunction with texture variables obtained with the GLCM method and wavelet analysis (as in [14]) does not significantly improve compared with ReUse with raw bands. This suggests that such a deep approach for AGB prediction can avoid the feature-engineering phase. It is worth noting that errors reported for the competitors are higher than those reported in the corresponding papers. This is because the ground truth used in this paper comes from ESA, while the original works used field measurements (not released for reproducibility purposes).

### 4.1. Case Study: Astroni Nature Reserve

A case study for the Astroni nature reserve in southern Italy was presented to show how ReUse, Sentinel-2, and ESA’s AGB public data can help estimate CO${}_{2}$ in forest areas and monitor deforestation downstream of events such as fires. ReUse is adopted with raw Sentinel-2 bands alone without using other extracted features that would not bring decisive benefits, as demonstrated in the previous section.

The area of Central Europe contained in the file ‘N60E00’ was chosen to monitor the Astroni reserve to train ReUse because it is certainly an area with characteristics closer to those of southern Italy than the other two datasets containing the areas of Vietnam and Myanmar. In [38], research was conducted within the Astroni Crater World Wildlife Fund (WWF) Reserve in the volcanic area of the Campi Flegrei in the urban area of Naples, Italy. The reserve (247 hectares) lies within the caldera of an extinct volcano, with a maximum altitude of 255 m above sea level and an elliptical shape (2 × 1.6 km). The inner part of the crater has a deep depression containing a lake, where a minimum altitude of 9 m above sea level is reached. In the central part of the crater, near the largest lake, two other small lakes and three hills rise to the bottom at 45, 74, and 82 m above sea level, respectively. Throughout the crater, [38] focused on the area of Holm oak forest (127 ha) and mixed forest (104 ha) to define the two main ecosystems and found that the total carbon stocks of the phytomass of these two ecosystems was 22,173 ± 7054 tons using sampling from April to October 2016.

**Figure 7:**
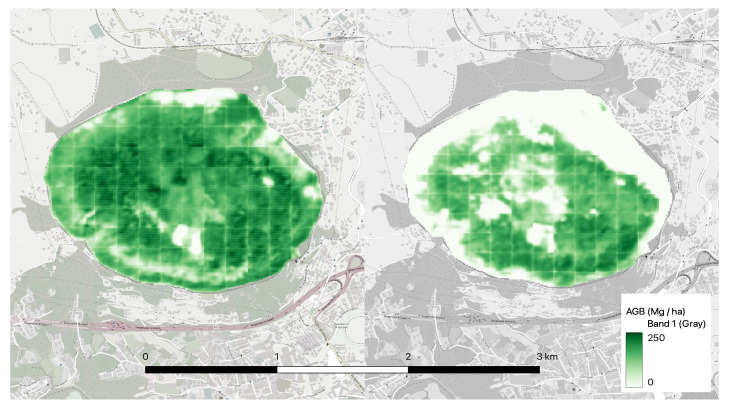
On the left is the predicted above-ground biomass raster of the Astroni nature reserve before the July 2017 fire; on the right is the predicted above-ground biomass raster after a major fire event for the same area.

In order to make inferences, the Sentinel-2 images downloaded on 31 May 2017 were upscaled to a spatial resolution of 10 m, assuming that the spatial correlations learned from the network at 100 m are also reproducible at 10 m; this improves the resolution of the predictions compared with the resolution of the AGB raster of the ESA CCI Biomass project, which is at 100 m. The predictions of carbon stocks were made by creating the predicted raster of AGB, namely ten by ten non-overlapping patches corresponding to one hectare each. Then, for each patch, the average AGB value expressed in tons per hectare was taken, which, when multiplied by 1 hectare, which is the extent of the patch, yields a value in tons. These values were summed over all patches, and the final result was multiplied by 0.5 [4,5] to obtain the value of absorbed carbon in tons. From this procedure, the estimate of the carbon stock for Astroni on 31 May 2017 was 18,748 tons, in line with the forecast of [38], which is 22,173 ± 7054 tons for phytomass in the year 2016. Please note that the latter includes the roots of the plants, which is not included in our estimate but which can be considered around 30% of above-ground biomass for temperate oak forest [39]. Thus, considering only the 0.3 × 18,748 + 18,748 estimates, we obtain a final value of 24,372 tons, which is within the range of Astroni’s estimate of 22,173 ± 7054 tons. In order to confirm the fact that the trained network recognizes a decrease in AGB downstream of a fire, the AGB raster of Astroni on 24 August 2017 downstream of the fire on July 2017 is shown in Figure 7 compared with the AGB raster of the same area before the fire. The estimated above-ground biomass carbon stock on 24 August 2017 for the nature reserve downstream of the fire on July 2017 is 10,104 tons, confirming the above.

It is stressed that the work performed is based entirely on open data, and the fact that a prediction based on public data is in agreement with ranges given by a ground truth gives worth to a tool that can be obtained without field measurements and that could be useful for monitoring carbon stocks in forest areas. The prediction after the fire cannot be verified in any way; however, the fact that the prediction before the fire is in line with the ground truth and that the forecast after this event shows a decrease due to the fire is encouraging.

## 5. Discussion with Conclusions

Sustainable Development Goal 15 aims to ‘protect, restore and promote the sustainable use of terrestrial ecosystems, sustainably manage forests, combat desertification, halt and reverse land degradation and halt biodiversity loss’ [3]. Furthermore, REDD+ projects aim to reduce greenhouse gases (GHG) concentrations in the atmosphere and contribute to climate change mitigation through various activities, including carbon stock enhancement [40]. Developing systems that can estimate the carbon absorbed by forests globally and monitor losses associated with deforestation phenomena, such as fires, in a timely fashion is essential. As explained in Section 1, systems that rely on field measurements are the most reliable. However, this work aimed to show how it is possible to estimate the carbon absorbed by forests and nature reserves such as Astroni using open AGB data from the ESA CCI Biomass Project in conjunction with Sentinel-2 images. To the best of our knowledge, this is the first time such a study has been performed by totally relying on open data only, without field measurements and using a deep approach based on a UNet architecture.

The proposed ReUse architecture is based on a pixel-wise regressive UNet, able to generate a pixel mask of AGB predictions with computational advantages, particularly when monitoring large areas. This is provides a great advantage over classical machine learning algorithms that require feature extraction work to derive indices that capture both spectral and spatial information content [10,14], or over solutions based on convolutional neural network approaches to estimate AGB using the commercial Worldview-2 satellite and visible spectrum images captured by an unmanned aerial vehicle [15,16], which produce a single value as a prediction of AGB. The computational advantage of UNet over simple CNNs lies in the fact that with CNNs, for each input pixel, its neighbourhood and associated bands are exploited to produce a single prediction of AGB, whereas with UNet, a patch of input pixels is associated with a patch of output pixels (e.g., for 16×16 pixels patches as in our case, 16 × 16 inferences with a simple CNN would be equivalent to a single inference with our UNet architecture).

ReUse, both when using raw bands only and raw bands in combination with spectral indices and texture features, showed better cross-validation performance in terms of MAE, RMSE, and $R^{2}$ against the two considered competitors [10,14] on three different areas (Table 2). It should also be noted that using a ReUse version leveraging raw bands together with some features does not lead to substantial advantages in terms of result accuracy compared with ReUse with raw bands alone, thus demonstrating that this approach can dispense with the feature-engineering phase and work directly with the appropriately normalized Sentinel-2 raw bands. When trained on the Central European zone, the network’s predictions were also validated on Astroni, a WWF reserve located close to the metropolitan area of Naples in southern Italy. The predicted carbon storage of above-ground biomass of Astroni prior to the July 2017 fire is 18,748 tons, in line with the estimates reported in [38]. In addition, an estimate of the carbon storage of the above-ground biomass of Astroni was also made on 24 August 2017 after a major fire event resulting in 10,104 tons. This last forecast cannot be verified in any way; however, the fact that the first prediction is in line with the ground truth and the second forecast shows a decrease due to the fire is encouraging.

In conclusion, the combined use of Sentinel-2 data and ESA’s AGB data with a UNet approach could be suitable for estimating the carbon absorbed in urban and rural areas and help monitor deforestation events without field measurements. To this aim, we have released the code to be used as a monitoring tool for deforestation. Future research directions could concern the use of multi-temporal Sentinel-2 data, in which spectral images of several dates are examined; the joint use of Sentinel-2 and Sentinel-1 as input for ReUse; or the application of super-resolution to improve the estimations of AGB and carbon stock in particular in urban areas. We will also explore other case studies to further verify how suitable the AGB data of ESA’s Climate Change Initiative Biomass project is as ground truth for AI-based models for forest monitoring.

## Figures and Tables

**Table 1:** List of the file names downloaded from the ESA Biomass Climate Change Initiative of the global above-ground forest biomass for 2018, v3, in Geotiff format containing AGB rasters. In each GeoTiff file, a study area was cut out and reported in well-known text (WKT) format.

AGB File Name	Area of Interest Represented in WKT
N60E00	Polygon ((6.41116296045328227 50.733179027500789, 7.47810311940281469 50.733179027500789, 7.47810311940281469 51.57252282701965385, 6.41116296045328227 51.57252282701965385, 6.41116296045328227 50.733179027500789))
N20E100	Polygon ((107.10766723771612874 12.51511413035953346, 107.83207927588165376 12.51511413035953346, 107.83207927588165376 13.26250192346686596, 107.10766723771612874 13.26250192346686596, 107.10766723771612874 12.51511413035953346))
N30E90	Polygon ((96.00143548690360262 22.97297258442770485, 96.49905818628494103 22.97297258442770485, 96.49905818628494103 23.42872411816529876, 96.00143548690360262 23.42872411816529876, 96.00143548690360262 22.97297258442770485))

**Table 2:** The results of the experiments performed on Vietnam and Myanmar in the study areas of [10,14] and our Central Europe study area. At each iteration of the eight-fold cross-validation, six folds are used for training, one for validation, and one for testing. The averages and standard deviations of the Mean Absolute Error (MAE), Root Mean Square Error (RMSE), and $R^{2}$ metrics are calculated on the test set. In bold: the best value for each metric per study area.

Area	Model	MAE	RMSE	$R^{2}$
Vietnam	ReUse with raw bands ReUse with feature extraction Competitor 1 [10] Competitor 2 [14]	**42.0** ± **6.6** 44.4 ± 6.0 60.1 ± 8.3 58.9 ± 8.6	**57.7** ± **7.3** 59.5 ± 4.7 73.0 ± 9.4 72.0 ± 9.7	**0.4** ± **0.2** **0.4** ± **0.2** 0.2 ± 0.2 0.2 ± 0.2
Myanmar	ReUse with raw bands ReUse with feature extraction Competitor 1 [10] Competitor 2 [14]	10.8 ± 2.0 **10.7** ± **2.2** 15.7 ± 1.9 15.5 ± 1.5	15.0 ± 2.4 **14.9** ± **2.6** 20.2 ± 2.3 20.1 ± 1.8	**0.7** ± **0.1** **0.7** ± **0.1** 0.4 ± 0.1 0.4 ± 0.1
Europe	ReUse with raw bands ReUse with feature extraction Competitor 1 [10] Competitor 2 [14]	24.5 ± 3.3 **24.1** ± **3.4** 32.5 ± 3.1 34.8 ± 3.1	**46.6** ± **5.2** 46.9 ± 4.2 48.0 ± 4.4 51.1 ± 3.9	**0.6** ± **0.1** **0.6** ± **0.1** 0.5 ± 0.5 0.5 ± 0.5

## Data Availability

Sentinel-2 L2A data are available at https://scihub.copernicus.eu/, accessed on 1 March 2023; ESA’s AGB data are available at https://climate.esa.int/en/projects/biomass/, accessed on 1 March 2023.
